# Resilient Leaf Physiological Response of European Beech (*Fagus sylvatica* L.) to Summer Drought and Drought Release

**DOI:** 10.3389/fpls.2018.00187

**Published:** 2018-02-19

**Authors:** Ellen E. Pflug, Nina Buchmann, Rolf T. W. Siegwolf, Marcus Schaub, Andreas Rigling, Matthias Arend

**Affiliations:** ^1^Forest Dynamics, Swiss Federal Institute for Forest, Snow and Landscape Research, Birmensdorf, Switzerland; ^2^Institute of Agricultural Sciences, ETH Zurich, Zurich, Switzerland; ^3^Laboratory of Atmospheric Chemistry, Paul Scherrer Institute, Villigen, Switzerland; ^4^Physiological Plant Ecology, University of Basel, Basel, Switzerland

**Keywords:** δ^13^*C*, non-structural carbohydrates, photosynthesis, recovery, water shortage

## Abstract

Drought is a major environmental constraint to trees, causing severe stress and thus adversely affecting their functional integrity. European beech (*Fagus sylvatica* L.) is a key species in mesic forests that is commonly expected to suffer in a future climate with more intense and frequent droughts. Here, we assessed the seasonal response of leaf physiological characteristics of beech saplings to drought and drought release to investigate their potential to recover from the imposed stress and overcome previous limitations. Saplings were transplanted to model ecosystems and exposed to a simulated summer drought. Pre-dawn water potentials (ψ_pd_), stomatal conductance (*g*_S_), intercellular CO_2_ concentration (*c*_i_), net-photosynthesis (*A*_N_), PSII chlorophyll fluorescence (*PI*_tot_), non-structural carbohydrate concentrations (*NSC*; soluble sugars, starch) and carbon isotope signatures were measured in leaves throughout the growing season. Pre-dawn water potentials (ψ_pd_), *g*_S_, *c*_i_, *A*_N_, and *PI*_tot_ decreased as drought progressed, and the concentration of soluble sugars increased at the expense of starch. Carbon isotopes in soluble sugars (δ^13^*C*_S_) showed a distinct increase under drought, suggesting, together with decreased *c*_i_, stomatal limitation of *A*_N_. Drought effects on ψ_pd_, *c*_i_, and *NSC* disappeared shortly after re-watering, while full recovery of *g*_S_, *A*_N_, and *PI*_tot_ was delayed by 1 week. The fast recovery of *NSC* was reflected by a rapid decay of the drought signal in δ^13^*C* values, indicating a rapid turnover of assimilates and a reactivation of carbon metabolism. After recovery, the previously drought-exposed saplings showed a stimulation of *A*_N_ and a trend toward elevated starch concentrations, which counteracted the previous drought limitations. Overall, our results suggest that the internal water relations of beech saplings and the physiological activity of leaves are restored rapidly after drought release. In the case of *A*_N_, stimulation after drought may partially compensate for limitations on photosynthetic activity during drought. Our observations suggest high resilience of beech to drought, contradicting the general belief that beech is particularly sensitive to environmental stressors.

## Introduction

Climate models predict an increase in the annual temperature in Central Europe by 2.7–4°C and a decrease in summer precipitation by 21–28% toward the end of the 21^st^ century (CH2011, 2011; IPCC, 2012). Additionally, climate variability is expected to increase, resulting in a higher frequency and intensity of extreme weather events, such as severe droughts, heavy rains and extraordinary cold or heat waves (Meehl and Tebaldi, 2004). This change in climate conditions may have a large impact on the physiological constitution of forest trees, thereby changing the productivity and composition of forest ecosystems (Ciais et al., 2005; Milad et al., 2010; Hanewinkel et al., 2012). Tree species that are less tolerant to drought will face a disproportionate risk of habitat loss due to impaired competitiveness compared to drought tolerant tree species (Ohlemüller et al., 2006; Czúcz et al., 2011). This may particularly apply to European beech (*Fagus sylvatica* L.), a major tree species in Central European forests, which is thought to be severely threatened by an increasing frequency and intensity of drought conditions (Rennenberg et al., 2004; Gessler et al., 2007; Kramer et al., 2010). There is, however, an ongoing debate among sylviculturists and tree biologists about the consequences of climate change for European beech (Ammer et al., 2005; Schütz, 2009).

The main processes involved in the drought response of European beech and other temperate tree species, as well as potential underlying mechanisms, have been studied intensively. The majority of these studies have demonstrated that drought influences various processes, such as photosynthesis, growth, hydraulic function and metabolism (e.g., Tognetti et al., 1995; Leuschner et al., 2001; Peuke et al., 2002; Leuzinger et al., 2005; Nahm et al., 2006; Weber et al., 2013; Aranda et al., 2015; Pflug et al., 2015). As a moderate anisohydric species, beech has the ability to adjust stomatal conductance (*g*_S_) and therefore can optimize photosynthetic assimilation rates (*A*_N_) under optimal or mild drought conditions (Tardieu and Simonneau, 1998; Peuke et al., 2002). However, this response can be disadvantageous under severe drought, when cavitation of the hydraulic system can occur, followed by increasing cellular water loss and damage to metabolically active leaf organs. The critical cavitation resistance of beech, where the risk of irreversible hydraulic dysfunction occurs, is comparable to that of other co-occurring tree species and thus does not support an exceptional sensitivity of European beech to drought (water potential -2.8 to -3.2 MPa; Wortemann et al., 2011; Choat et al., 2012; Gleason et al., 2016). Dendroecological studies, however, suggest a high sensitivity of European beech to soil water shortage (Dittmar et al., 2003; Gessler et al., 2004), which is in line with the ecological preference of this species for mesic soils with a sufficient water supply (Peters, 1997; Ellenberg, 2009) and supports the general view of European beech as a drought sensitive tree species.

In contrast to the vast number of studies reporting instantaneous tree responses to drought, little is known about recovery from this environmental stress, even though this ability is an important factor determining the drought resistance of trees on a longer time scale. A few studies have considered the photosynthetic response of saplings exposed to cycles of drought and re-watering (Tognetti et al., 1995; Gallé and Feller, 2007; Arend et al., 2013, 2016b; Blessing et al., 2016), while others have focused on the year-to-year variation in tree-ring growth in adult trees caused by annual fluctuations in precipitation (Dittmar et al., 2003; Pretzsch and Dieler, 2011). Findings from these studies have demonstrated the ability of beech to resume physiological activity, particularly photosynthesis, and growth after severe drought, but the processes occurring after stress release remain poorly understood. Zang et al. (2014) assessed dynamics and patterns of carbon allocation in potted beech saplings under dry and rewetted soil conditions. This experiment not only showed that beech saplings recovered quickly from severe drought but also provided first insights into the different uses of recent photo-assimilates under severe drought and shortly after rewetting. Furthermore, other recent studies have indicated a close coupling of respiratory sink activity in the roots and rhizosphere with photo-assimilation after drought release (Blessing et al., 2016). However, few experiments have followed drought-rewetting responses over a whole growing season, even though it is clear that seasonal aspects of drought development and drought release should not be ignored. In fact, recent studies have shown that trees not only recover from drought but even exhibit increased physiological activity in terms of photosynthesis to compensate for the limitations imposed by previous drought (Arend et al., 2016b; Hagedorn et al., 2016).

The limited knowledge of drought effects over longer time scales, particularly processes occurring after drought release, prevents us from predicting tree responses to future environmental conditions characterized by greater variability of precipitation and thus alternating drought and recovery periods (Reichstein et al., 2013). In this study, we followed the seasonal response of leaf physiological characteristics of beech to a simulated summer drought and drought release. More specifically, we subjected transplanted saplings in large outdoor model ecosystems to a slowly developing soil water shortage and subsequent re-watering. Leaf physiological changes were monitored with weekly to biweekly measurements of pre-dawn leaf water potential (ψ_pd_), stomatal conductance (*g*_S_), intercellular CO_2_ concentration (*c*_i_), net-photosynthesis (*A*_N_) and PSII fluorescence (*F*_v_/*F*_m_; *PI*_tot_), together with an analysis of non-structural carbohydrate concentrations (*NSC*; soluble sugars and starch) and the carbon isotopic composition in bulk leaf material (δ^13^*C_L_*) and soluble sugars (δ^13^*C*_S_). With this experimental setup, we aimed to understand the temporal course of the drought response and the recovery process after drought release. We specifically addressed the following questions: (i) at what drought stress intensity (ψ_pd_) is a leaf physiological drought response detectable, (ii) how fast does leaf physiological activity recover after drought release, and (iii) is there a drought effect imprinted on subsequent leaf physiological activity?

## Materials and Methods

### Experimental Design and Plant Material

European beech (*Fagus sylvatica* L.) saplings 10–20 cm tall and 3–5 years old were collected from 12 beech populations (Arend et al., 2016a) and transplanted to the 16 lysimeter plots of the outdoor model ecosystems at the Swiss Federal Research Institute WSL (47°21′ N, 8°27′ E, 545 m a.s.l.). In each plot, two saplings from each population were grown with a randomized distribution. The plots are 3 m^2^ in area, have a depth of 150 cm and are filled with natural forest soil (acidic Haplic Alisol; loamy sand; pH 4.6). The water regime of each plot is controlled by sliding glass roofs, which close automatically at the onset of rain fall, and an automated irrigation system. During the growing season, the plots were irrigated every second or third day with 50 l m^-2^ deionized water enriched with nutrients to simulate the average composition of ambient rainfall (Kuster et al., 2013). After 2 years, when the saplings had reached a height of approximately 150 cm, a summer drought was imposed in 8 of the 16 plots by omitting the irrigation from June to mid-August 2013, while the other eight plots were regularly irrigated. Leaf development was complete when the drought treatment started. After 10 weeks, the plots were intensely re-watered and afterward regularly irrigated. For this particular study, six control and six drought-treated saplings from a mesic beech population (Collombey, Switzerland; 46°16′ N 6°56′ E; annual precipitation 1,055 mm; annual temperature 8.9°C), each growing in a separate plot, were selected.

### Measurements of Soil Water Content and Pre-dawn Leaf Water Potential

Soil water content (*SWC*) was measured volumetrically at 30 cm soil depth using PC-controlled soil moisture probes (Decagon 5TM; Decagon, United States) installed in each plot of the model ecosystem. Pre-dawn leaf water potentials (ψ_pd_) were measured (bi)weekly using a Scholander pressure chamber (M 600; Mosler Tech Support, Berlin, Germany) in two leaves collected before sunrise from the outer part of the canopy of each sapling. To minimize the impact of the frequent leaf harvest on the saplings, the same leaf material was used for the extraction of *NSC*. The leaves were briefly microwaved to stop any metabolic activity and then oven-dried at 60°C for 48 h.

### Measurements of Leaf Gas Exchange and PSII Photochemistry

On every sampling date, leaf gas exchange characteristics, i.e., net-photosynthesis (*A*_N_), intercellular CO_2_ concentration (*c*_i_) and stomatal conductance (*g*_S_), of two to four sun-exposed leaves per sapling were measured between 11h00 and 16h00. Measurements were performed with a portable photosynthesis system using a broadleaf cuvette (LI-COR 6400; LI-COR, Lincoln, NE, United States). Conditions in the cuvette were controlled during the measurements to maintain a CO_2_ concentration of 400 ppm and a photon flux density of 1,000 μmol m^-2^ s^-1^, while temperature was adjusted to track values outside the cuvette, which ranged from 11.2 to 36.4°C during the measurements. Fast fluorescence kinetics were analyzed pre-dawn using a plant efficiency analyzer (Pocket PEA, Hansatech Instruments, Ltd., Norfolk, United Kingdom). These fluorescence measurements were conducted on each sampling date prior to the analysis of ψ_pd_ in the dark-adapted state. After a saturating light pulse of 3,500 μmol quanta m^-2^ sec^-1^ of red light (650 nm) was applied, the increase in fluorescence was recorded at a high resolution for 1 s. Maximum quantum efficiency of photosystem II (*F*_v_/*F*_m_) and the total performance index (*PI*_tot_) were calculated using PEA plus 1.10 (Hansatech Instruments, Ltd., Norfolk, United Kingdom).

### Quantification of Non-structural Carbohydrates

Non-structural carbohydrates (starch, glucose, fructose, and sucrose) were extracted according to Critchley et al. (2001). Approximately, 100 mg of oven-dried and powdered leaf material was incubated for 15 min with 1.12 M perchloric acid and then centrifuged at 3000 *g* for 15 min. The supernatant used for quantification of soluble sugars was adjusted to pH 6 by the addition of 2 M KOH, 0.4 M MES, and 4 M KCl. The precipitated potassium perchlorate was removed by centrifugation at 3000 *g* for 15 min. Sucrose in the extract was broken down to glucose and fructose by invertase (Roche, Rotkreuz, Switzerland). Free glucose and glucose originating from sucrose were then converted to gluconate-6-phosphate by glucose-6-phosphate dehydrogenase (Roche, Rotkreuz, Switzerland) and determined photometrically with a 96-well microplate reader (ELx800, BioTek, Luzern, Switzerland). Afterward, fructose was converted to glucose by phosphogluco-isomerase (Roche, Rotkreuz, Switzerland) and then measured as described above. For starch quantification, the remaining pellet was thoroughly washed with 80% EtOH, dried at room temperature and re-suspended in water before starch was broken down to glucose monomers via amyloglucosidase and α-amylase for 2 h at 37°C (both Roche, Rotkreuz, Switzerland) and then determined photometrically as described above. Photometric quantification was performed according to Hoch et al. (2003). The *NSC* concentrations are expressed on a dry matter basis (mg g^-1^).

### Extraction of Soluble Sugars for δ^13^C Analysis

Soluble sugars for δ^13^C analysis were extracted and prepared according to the method established by Wanek et al. (2001) and modified by Göttlicher et al. (2006) and Richter et al. (2009). Briefly, 100 mg of powdered leaf material was extracted with 1 mL MCW (methanol, chloroform, water, 12:3:5, v/v/v) for 30 min at 70°C. After cooling to room temperature, the sample was centrifuged at 10,000 *g* for 2 min, and 800 μL of the supernatant was used for the extraction of soluble sugars. Phase separation of the supernatant was induced by adding 800 μL water and 250 μL MCW and then vigorously mixing. After every six samples, one blank was processed (800 μL MCW). After centrifugation at 10,000 *g* for 2 min, 1.2 mL of the upper aqueous phase was mixed with 500 μL chloroform and centrifuged for phase separation. Then, 1 mL of the upper phase was oven-dried for 24 h at 60°C. The sample was re-dissolved in 1 mL water and separated using an ion-exchange cartridge (5 mL syringes, BD Plastipak^TM^, Beckon Dickinson S.A., Madrid, Spain) made of 2.2 mL cation-exchange resin (DOWEX 50W X 8, 50–100 mesh, H^+^-form) above 3.2 mL anion-exchange resin (DOWEX 1 X 8, 50–100 mesh, HCOO-form), separated by filter paper. After rinsing the columns with 30 mL water, 35 mL of the eluate, mainly consisting of soluble sugars, was collected. The samples were lyophilized and re-dissolved in 1 mL water. Volumes of 150 μL of each sample were pipetted into tin capsules and oven-dried for 48 h at 60°C before isotopic analysis was conducted.

### Measurement of δ^13^C in Leaf Bulk Tissue and Soluble Sugars

For the analysis of δ^13^C in leaves, about 0.5 mg of the powdered leaf material or extracted leaf sugars was weighed into tin capsules (Säntis Analytical, Teufen, Switzerland). The samples were combusted to CO_2_ with an excess of oxygen at 1,020°C in an elemental analyzer (EA-1110, Carlo Erba Thermoquest, Milan, Italy), which was connected to a Delta S mass spectrometer with a CONFLO II system (both Finnigan MAT, Bremen, Germany), performing in continuous flow mode. The isotope ratio of δ^13^C is given in reference to its international standard, Vienna Pee Dee Belemnite (VPDB), in the delta notation in ‰: δ_sample_ = (R_sample_/R_standard_-1), with R_sample_ being the ^13^C/^12^C ratio of the sample and R_standard_ being the ratio of VPDB. The standard deviation of laboratory cellulose standards was used as an estimate of analysis precision and was lower than 0.10‰.

### Statistical Analysis

All data were analyzed using SPSS 21.0 (SPSS, Inc., Chicago, IL, United States). In order to evaluate differences among the treated and the control saplings, a non-parametric test of variance (Mann–Whitney *U*-test) was applied and FDR (false discovery rate) corrected to account for multiple comparisons (Benjamini and Hochberg, 1995). Data were analyzed separately for the drought and recovery period to detect days with significant differences between the treatments. The differences between the treatments were considered significant when *P* ≤ 0.05. The statistical tests were performed with four to six replicates per treatment group.

## Results

### Soil Water Content and Leaf Water Potential

**FIGURE 1:**
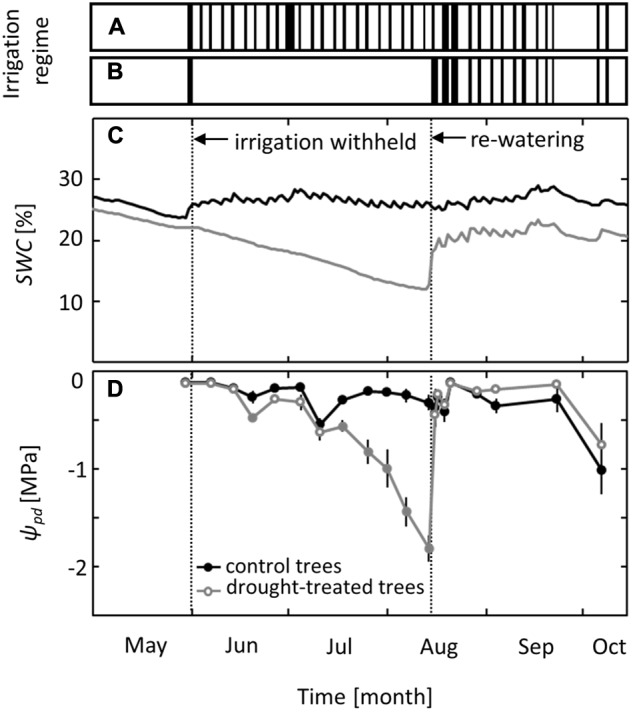
Irrigation regime and effects on soil water content (*SWC*) and pre-dawn leaf water potential (ψ_pd_). Timing of the irrigation events in **(A)** the control and **(B)** the drought treatment. **(C)** *SWC* at a depth of 30 cm and **(D)** ψ_pd_ in control and drought-treated plots. Filled gray circles indicate significant differences between the two treatments (*P* ≤ 0.05). For the drought treatment, the two vertical dotted lines represent the date when irrigation was first withheld and the date of the re-watering event. Data are means of *n* = 6; ±1 SE.

Irrigation was excluded from the drought-treated plots for 10 weeks, from June to mid-August, while control plots received regular irrigation (**Figures 1A,B**). Soil water content in the control plots ranged from 24 to 29% throughout the growing season (**Figure 1C**). In drought-treated plots, it ranged from 18 to 25% before and after treatment but decreased gradually to 12% during the drought period without irrigation. Trees in control plots maintained pre-dawn leaf water potentials (ψ_pd_) above -0.5 MPa, except at the end of the growing season in October, when leaf senescence started and ψ_pd_ decreased to values of -1.0 MPa (**Figure 1D**). In drought-exposed saplings, ψ_pd_ started to decrease in mid-July, 7 weeks after irrigation was first withheld, and reached a minimum value of -1.8 MPa (mean of six replicates) at the end of the drought period in mid-August. After the first re-watering event, ψ_pd_ increased within 1 day to the level observed for control trees and even reached slightly higher ψ_pd_ values than those of control trees (**Figure 1D**).

### Leaf Gas Exchange and PSII Photochemistry

**FIGURE 2:**
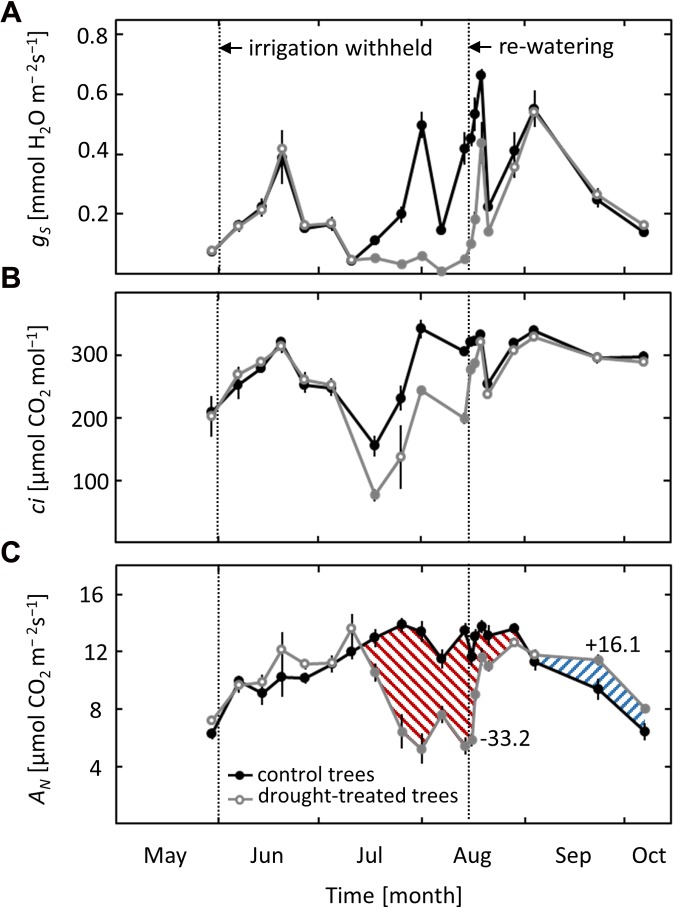
Seasonal course of leaf gas exchange. **(A)** Stomatal conductance (*g*_S_), **(B)** intercellular CO_2_ concentration (*c*_i_) and **(C)** net-photosynthesis (*A*_N_) in control and drought-treated beech saplings. Numerical data indicate the drought limitation and post-drought stimulation of *A*_N_ (difference of the integrated areas below the curves). Filled gray circles indicate significant differences between the two treatments (*P* ≤ 0.05). For the drought treatment, the two vertical dotted lines represent the date when irrigation was first withheld and the date of the re-watering event. Data are means of *n* ≥ 4; ±1 SE (11.07).

Due to variable weather conditions, with both hot, sunny days and cool, cloudy days, stomatal conductance (*g*_S_) varied strongly throughout the growing season, regardless of the applied drought treatment (**Figure 2A**). Nevertheless, a clear drought effect on *g*_S_ was observed in non-irrigated saplings. Stomatal conductance started to decrease when ψ_pd_ dropped below -0.6 MPa in mid-July and remained lower than values of the controls until re-watering in mid-August. After re-watering, *g*_S_ showed a fast initial increase and recovered fully after 6 days, reaching values comparable to those of controls. A similar pattern was obtained for intercellular CO_2_ concentration (*c*_i_), which decreased when ψ_pd_ dropped below -0.6 MPa and recovered quickly after drought release (**Figure 2B**).

Net-photosynthesis (*A*_N_) showed a distinct seasonal pattern, with higher rates in summer and lower rates in spring and autumn (**Figure 2C**). Net-photosynthesis in drought-exposed saplings was comparable to that in control trees as long as *ψ*_pd_ remained above -0.6 MPa. When ψ_pd_ dropped below this critical value in mid-July, *A*_N_ started to decrease and was reduced by up to 60% at the end of the drought treatment in mid-August (**Figure 2C**). Net-photosynthesis responded quickly to re-watering, reaching 85% of the photosynthetic rate in control trees after 4 days and recovering completely after 1 week. Afterward, previously drought-exposed saplings had higher values of *A*_N_ compared to control trees, with a post-drought stimulation apparent toward the end of September and the beginning of October. The overall limitation of *A*_N_ in drought-treated saplings in relation to control saplings (estimated from the difference of the integrated areas below the curves) was -33%. The post-drought stimulation, integrated over the whole period after full recovery, was +16%.

**FIGURE 3:**
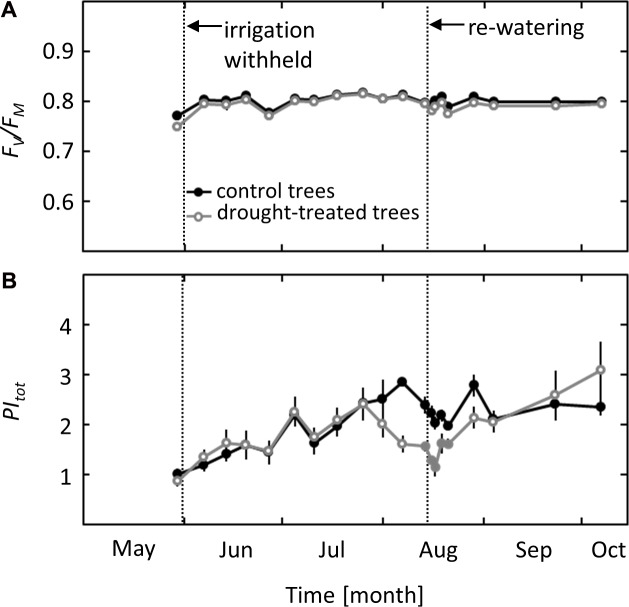
Seasonal course of pre-dawn PSII Chlorophyll Fluorescence parameters in control and drought-treated beech saplings. **(A)** Maximum quantum yield (*F*v/*F*m) and **(B)** total performance index (*PI*_tot_) in control and drought-treated saplings. Filled gray circles indicate significant differences between the two treatments (*P* ≤ 0.05). For the drought treatment, the two vertical dotted lines represent the date when irrigation was first withheld and the date of the re-watering event. Data are means of *n* = 6; ±1 SE.

Photochemical effects on photosynthesis were studied using PSII chlorophyll fluorescence analysis and the derived parameters maximum quantum yield of PSII (*F*_v_/*F*_m_) and total performance index of PSII (*PI*_tot_). While *F*_v_/*F*_m_ did not show any seasonal variation or response to the imposed drought, *PI*_tot_ showed a clear drought signal (**Figures 3A,B**). A decrease in *PI*_tot_ was observed in mid-August when ψ_pd_ reached values of -1.8 MPa, more than 3 weeks after the first drought effects on *g*_S_ and *A*_N_ were detected. After re-watering, *PI*_tot_ showed a delayed response, increasing slowly after 2 days and recovering completely 1 week after drought release, at the same time when full recovery of *A*_N_ was observed.

### Non-structural Carbohydrate Concentrations

**FIGURE 4:**
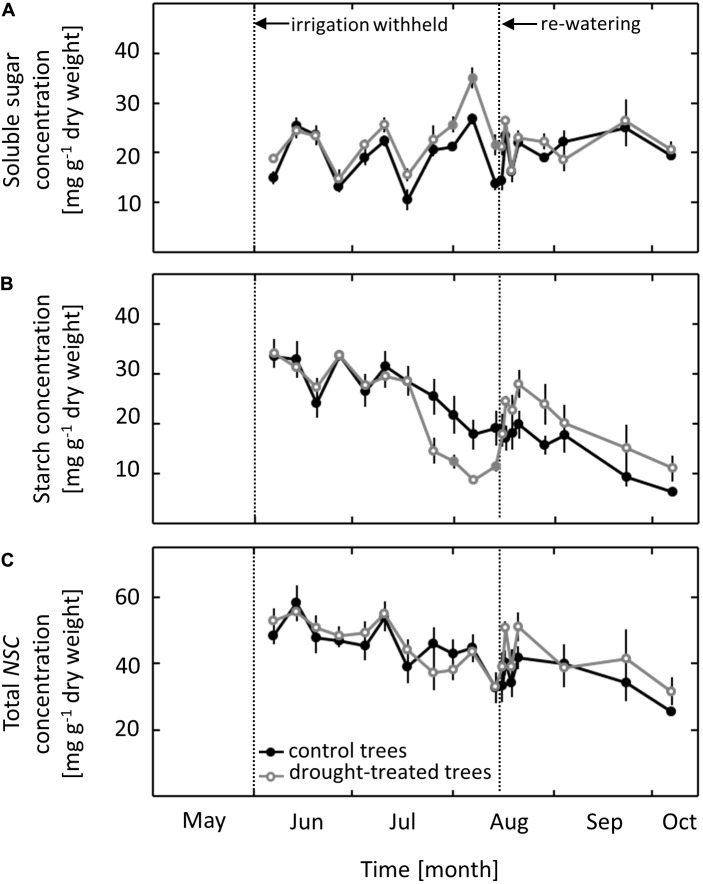
Seasonal course of non-structural carbohydrates (*NSCs*) in control and drought-treated beech saplings. **(A)** Soluble sugars **(B)** starch and **(C)** total concentrations of *NSCs*. Filled gray circles indicate significant differences between the two treatments (*P* ≤ 0.05). For the drought treatment, the two vertical dotted lines represent the date when irrigation was first withheld and the date of the re-watering event. Data are means of *n* ≥ 5; ±1 SE (starch content: 7 August, *n* = 3, ±1 SE).

Concentrations of *NSCs* were measured in pre-dawn harvested leaves. In control saplings, the concentration of soluble sugars ranged from 10 to 25 mg g^-1^ dry weight throughout the whole growing season, with no clear seasonal trend (**Figure 4A**). The concentration of starch and of total *NSC* ranged from 6 to 35 mg g^-1^ dry weight and 25 to 58 mg g^-1^ dry weight, respectively, throughout the growing season, with a gradual decline occurring from June to October (**Figures 4B,C**). While total *NSC* was not affected by the imposed drought, both starch and soluble sugars showed a distinct drought response. The starch concentration in leaves of drought-exposed saplings decreased as ψ_pd_ dropped to -1.0 MPa, 2 weeks after *g*_S_ and *A*_N_ showed a drought response. After re-watering in mid-August, the starch concentration increased within 1 day to the level in control saplings. After full recovery, the previously drought-treated saplings had slightly higher starch concentrations than control saplings, although this difference was not significant (**Figure 4B**). Soluble sugars showed a drought response that was the inverse to that observed for starch, with increasing concentrations during the drought treatment (**Figure 4A**). The first drought effects on the concentration of soluble sugars were observed when ψ_pd_ reached a value of -1.0 MPa. Soluble sugar concentrations returned to the same level as observed in control saplings within 1 day of re-watering and subsequently remained at the control level.

### ^13^C Signatures in Leaf Bulk Tissue and Sugars

**FIGURE 5:**
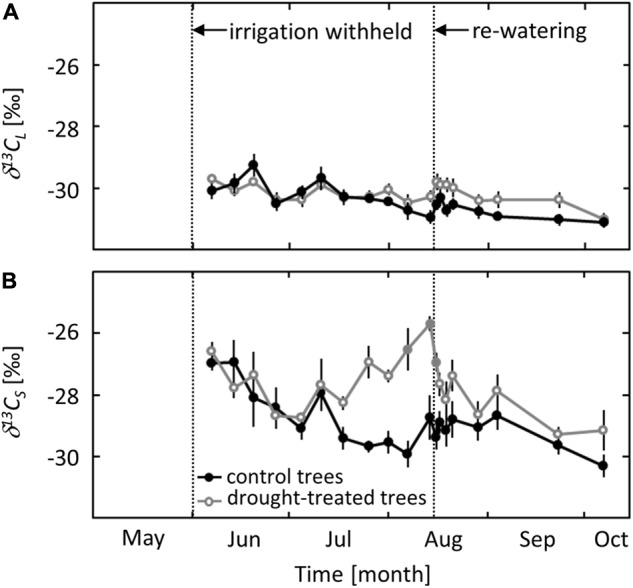
Seasonal changes in the isotopic signature of leaf bulk tissue (δ^13^*C_L_*, **A**) and sugars (δ^13^*C*_S_, **B**) in control and drought-treated saplings. Filled gray circles indicate significant differences between the two treatments (*P* ≤ 0.05). For the drought treatment, the two vertical dotted lines represent the date when irrigation was first withheld and the date of the re-watering event. Data are means of *n* = 6; ±1 SE (δ^13^*C*_S_: 01 and 07 August 2013, *n* = 4, ±1 SE).

The ^13^C signature of the leaf bulk tissue (δ^13^*C_L_*) did not show seasonal variation or a distinct drought response (**Figure 5A**). For both the control and the drought treatment, δ^13^*C_L_* was around -30‰ at the beginning of the measurements in early June and decreased gradually to -31‰ in early October.

In contrast to δ^13^*C_L_*, the ^13^C signature of soluble sugars (δ^13^*C*_S_) showed a clear seasonal trend and a distinct drought response. δ^13^*C*_S_ in control saplings decreased gradually from -27‰ in June to -30‰ in early October. In drought-exposed saplings, an (non-significant) increase of δ^13^*C*_S_ by 2.7‰ (*P* = 0.06) was observed when ψ_pd_ reached a value of -0.8 MPa (**Figure 5B**). δ^13^*C*_S_ increased further with decreasing ψ_pd_ and reached a maximum value of -26‰ at the end of the drought period in mid-August. The drought signal in δ^13^*C*_S_ declined quickly after re-watering, reaching values close to those in control saplings after 1 day.

## Discussion

### Responses to Drought

**FIGURE 6:**
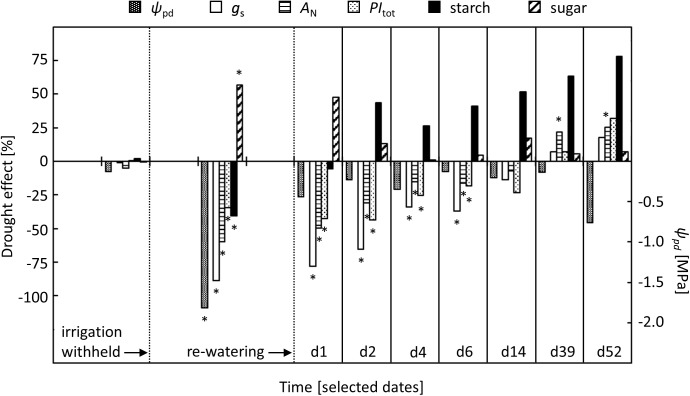
Comparison of relative drought effects on stomatal conductance (*g*_S_), net-photosynthesis (*A*_N_), total performance index of PSII (*PI*_tot_), and concentrations of starch and sugars in relation to changes in pre-dawn leaf water potential (ψ_pd_) in the drought-treated saplings. Effects are shown for pre-drought, drought, and re-watering conditions. For re-watering conditions, specific days after the first re-watering event (d1, d2, …, d52) were selected. Asterisks indicate significant differences between the two treatments (*P* ≤ 0.05). For the drought treatment, the two vertical dotted lines represent the date when irrigation was first withheld and the date of the re-watering event.

Our study on beech saplings revealed a sequence of leaf physiological changes that gradually developed in response to increasingly severe drought conditions (**Figure 6**). These responses were related to pre-dawn leaf water potential (ψ_pd_), a physiological indicator of plant-internal water relations (Cochard et al., 2001; Ehrenberger et al., 2012). Leaf physiological responses were observed first for stomatal conductance (*g*_S_), intercellular CO_2_ concentration (*c*_i_) and net-photosynthesis (*A*_N_), which started to decrease when ψ_pd_ dropped below -0.6 MPa. Previous studies on beech showed similar responses of *g*_S_ and *A*_N_ to decreasing ψ_pd_, suggesting that a ψ_pd_ of around -0.6 MPa represents a general threshold for drought effects on beech leaf gas exchange (Aranda et al., 2012; Cocozza et al., 2016).

Shortly after *A*_N_ started to decrease, the isotopic signature of soluble sugars (δ^13^*C*_S_) showed an increase in drought-treated saplings relative to values in control saplings. This result may indicate limitations of *A*_N_, due either to high leaf resistance to CO_2_ diffusion and/or to impaired photochemistry (Farquhar et al., 1989). The latter explanation can be excluded, as chlorophyll fluorescence did not indicate impaired photochemistry and photochemical reactions are resistant to moderate stress (Kaiser, 1987; Havaux, 1992; Epron and Dreyer, 1993; Arend et al., 2013). Thus, the early decrease of *A*_N_ must be a result of limited CO_2_ diffusion to the sites of photosynthetic reactions in the chloroplasts. Indeed, diffusional limitations are the predominant factors controlling photosynthesis in response to stress, including changes in both stomatal and mesophyll resistance (Centritto et al., 2003, 2009; Galmés et al., 2007; Flexas et al., 2008). In the present study, however, we obtained evidence that high stomatal resistance, i.e., low *g*_S_, was the rate-limiting factor for *A*_N_. In fact, the decrease in *A*_N_ was accompanied by a parallel decline in *g*_S_ and *c*_i_, suggesting that CO_2_ diffusion through the stomata was more affected than subsequent CO_2_ diffusion through the mesophyll. This interpretation is consistent with the positive relationship between mesophyll resistance and *c*_i_ (Flexas et al., 2008), which in turn supports our assumption that stomatal resistance was the rate-limiting factor in leaf CO_2_ diffusion. Reduced *c*_i_ has previously been shown to be an early response to water stress and is exclusively related to stomatal limitation of photosynthesis (Sharkey and Seemann, 1989; Grassi and Magnani, 2005). Overall, the initial increase in δ^13^*C*_S_ in drought-exposed beech most likely reflects stomatal limitation of *A*_N_, which also suggests higher photosynthetic water use efficiency (Farquhar et al., 1989). With increasingly severe drought, however, when ψ_pd_ decreased to -1.8 MPa, concurrently measured chlorophyll fluorescence suggested additional limitations of photochemical reactions. The total performance index of PSII (*PI*_tot_), a stress-sensitive fluorescence parameter (Strasser et al., 2010; Albert et al., 2011), indicated impaired PSII photochemistry that may have contributed to the further decline in *A*_N_ with drought.

Impaired photosynthesis, as observed in the present study, cause concomitant changes in leaf carbon metabolism (Pinheiro and Chaves, 2011). Reduced availability of recent photo-assimilates, for instance, may lead to greater consumption of transiently stored starch as an alternative source of sugars for metabolic activity, osmoregulation, and cellular stress defense (Chaves, 1991; Hare et al., 1998). In drought-exposed beech, we observed an increase in the concentration of soluble sugars at the expense of starch when drought stress severity increased, as indicated by a decline in ψ_pd_ to -1.0 MPa. We therefore cannot exclude that carbon partitioning from starch to sugars may have contributed to the observed increase in δ^13^*C*_S__,_ as sugars derived from transiently stored starch are enriched in ^13^C relative to sugars from recently assimilated carbon (Rossmann et al., 1991; Gleixner and Schmidt, 1997; Gleixner et al., 1998). Indeed, starch concentrations started to decrease when δ^13^*C*_S_ increased, while the isotopic signature of leaf bulk material (δ^13^*C_L_*) was less affected. Thus, there remains some uncertainty as to the source of increased δ^13^*C*_S_, which seemingly compromises the interpretation given above linking δ^13^*C*_S_ with a diffusional limitation of *A*_N_. Transient starch, however, forms a highly dynamic carbon pool that relies on the daily uptake and release of recently assimilated carbon. The rapid turnover of starch explains why sugars derived from transiently stored starch integrate the same physiological signals into their isotopic signature as sugars derived from recently assimilated carbon (Brugnoli et al., 1988; Blessing et al., 2015). It is therefore very likely that the increase in δ^13^*C*_S_ still reflects diffusional limitation of *A*_N_, regardless of the fact that different sugar fractions contribute to this signal. Despite altered sugar and starch concentrations, the total amount of leaf *NSC* was not affected during drought. Thus, we can exclude the possibility that carbon starvation plays a role in the drought response of beech, which is in line with findings from previous studies on drought-exposed beech and oak trees (Li et al., 2013; Liu et al., 2017).

### Responses after Drought Release

An important, but not fully explored, aspect of drought tolerance in trees is their ability to recover after drought release and quickly resume full physiological activity (Gallé and Feller, 2007; Arend et al., 2013, 2016b). In the present study, we took advantage of the immediate restoration of tree internal water relations after re-watering, which allowed us to study the recovery of leaf physiological activities without interfering effects of further plant-hydraulic constraints (**Figure 6**). Indeed, after the first re-irrigation pulse was applied, ψ_pd_ increased within 1 day to the level of control trees, even though soil re-wetting was not complete after the long-lasting summer drought. From this observation, we conclude that the hydraulic system in beech is fairly resistant toward drought-induced damage, as suggested in previous studies (Wortemann et al., 2011; Choat et al., 2012; Gleason et al., 2016). This interpretation is in line with previous observations that critical values of ψ_pd_, which induce recovery failure of tree internal water relations, photosynthesis and other eco-physiological traits, are notably low in beech compared with other angiosperm tree species (Urli et al., 2013).

The immediate restoration of tree internal water relations was followed by a rapid adjustment of the leaf carbon metabolism to that in controls, as indicated by increasing starch and decreasing sugar levels and a fast decay of the drought signal in δ^13^*C*_S_. The latter result suggests a rapid turnover of primary carbon metabolites after drought release, presumably driven by the increased delivery of new photo-assimilates and by an enhanced carbon demand for leaf respiration and export of sugars to respiratory sinks in the roots and rhizosphere (Zang et al., 2014). In fact, leaf carbon metabolism depends on whole tree carbon allocation dynamics, as demonstrated by a close coupling of above and belowground carbon fluxes (Högberg et al., 2001, 2008; Epron et al., 2011). In beech, it has recently been proposed that the drought recovery of respiratory sink activity in the roots and rhizosphere triggers the carbon export from leaves, which implies a lower residence time of recent assimilates in leaves and thus rapid turnover of primary carbon metabolites (Hagedorn et al., 2016).

Although levels of sugars and starch recovered within 1 day, photosynthetic traits remained constrained by the previous drought. After an initial rise, *A*_N_ did not increase further and remained for 1 week at lower levels than in controls. Other studies on beech have reported different time scales for a full recovery of *A*_N_, ranging from 3 days to 4 weeks (Gallé and Feller, 2007; Zang et al., 2014; Blessing et al., 2016). However, results of drought experiments are difficult to compare, owing to the heterogeneity of experimental conditions, different stress intensities and ecological diversity of the plant material (Miyashita et al., 2005; Vicca et al., 2012; Arend et al., 2016b). In our study, the post-drought limitation of *A*_N_ was attributable to a persistent drought effect on stomatal and photochemical traits of photosynthesis, i.e., *g*_S_ and *PI*_tot_, but we cannot exclude that altered mesophyll conductance was also involved (Flexas et al., 2008; Centritto et al., 2009). While *g*_S_ responded quickly to the first re-watering pulse, it did not fully recover and instead remained at lower levels than in controls for approximately 1 week. In contrast, *PI*_tot_ responded much more slowly to the first re-watering pulse, and full recovery was delayed by 1 week, indicating a down-regulation of PSII photochemistry that may have contributed to the delayed recovery of *A*_N_.

There is increasing evidence that a drought effect is imprinted on trees, which compensates for the previous limitations of metabolic activities (Arend et al., 2016b; Hagedorn et al., 2016). Together with the tree’s capacity to recover from limiting stress, such a post-drought effect may be crucial for the tree’s overall drought resilience. In support of this, we observed a slight, but significant, post-drought stimulation of *A*_N_, which counteracted the previous limitation of *A*_N_ under drought conditions. Interestingly, *A*_N_ started to increase after *PI*_tot_ recovered completely, suggesting that the post-drought stimulation of *A*_N_ depends directly or indirectly on full PSII photochemical activity. Altered stomatal regulation could be excluded as a cause for the observed post-drought stimulation of *A*_N_, as *g*_S_ and *c*_i_ did not show any further change in previously drought-exposed beech saplings after full recovery. Instead, we observed a distinct trend toward increased starch concentrations and higher *PI*_tot_ values in previously drought-exposed beech saplings, lending further indirect support for improved leaf metabolic activity after drought recovery.

## Conclusion

In this study, we investigated the response of leaf physiological traits in beech to a simulated summer drought, not only describing the well-known limitations of these traits imposed by sudden drought but also highlighting the fast recovery of tree internal water relations and carbon metabolism and demonstrating a post-drought stimulation of photosynthesis after drought release. This last finding should be investigated further in future studies to better understand the underlying mechanisms and to evaluate the implications of stimulated photosynthesis for tree and ecosystem carbon fluxes under future climate conditions with greater variability of precipitation and thus alternating drought and recovery periods. Overall, the present study suggests a relatively high resilience of European beech to summer drought that needs to be considered in models predicting the impact of climate change on species distribution and ecosystem functioning.

## Author Contributions

EP, RS, MS, and MA conceived and designed the experiments. EP, RS, and MA performed the experiments and analyzed the data. EP, NB, and MA wrote the manuscript. AR provided the editorial advice.

## Conflict of Interest Statement

The authors declare that the research was conducted in the absence of any commercial or financial relationships that could be construed as a potential conflict of interest.
